# Different effects of acute and chronic oxidative stress on the intestinal flora and gut-liver axis in weaned piglets

**DOI:** 10.3389/fmicb.2024.1414486

**Published:** 2024-06-17

**Authors:** Hongyu Zhang, Xuan Xiang, Chenyu Wang, Tiejun Li, Xuping Xiao, Liuqin He

**Affiliations:** ^1^Department of Otorhinolaryngology Head and Neck Surgery, The First Affiliated Hospital of Hunan Normal University (Hunan Provincial People’s Hospital), Changsha, China; ^2^Hunan Provincial Key Laboratory of Animal Intestinal Function and Regulation, Laboratory of Animal Nutrition and Hunan Health, College of Life Sciences, Hunan Normal University, Changsha, China; ^3^Hunan Provincial Key Laboratory of Animal Nutritional Physiology and Metabolic Process, CAS Key Laboratory of Agro-Ecological Processes in Subtropical Region, National Engineering Laboratory for Pollution Control and Waste Utilization in Livestock and Poultry Production, Institute of Subtropical Agriculture, Chinese Academy of Sciences, Changsha, China

**Keywords:** acute oxidative stress, chronic oxidative stress, gut-liver axis, intestinal flora, weaned piglets

## Abstract

**Introduction:**

Oxidative stress plays a pivotal role in modulating the balance of intestinal flora and the gut-liver axis, while also serving as a key determinant of the growth potential of weaned piglets. However, few studies have subdivided and compared acute and chronic oxidative stress.

**Methods:**

In this study, an intestinal model of acute oxidative stress in weaned piglets using paraquat (PQ) and a chronic oxidative stress model using D-galactosa in weaned piglets were conducted. And we further systematically compare their effects.

**Results:**

Both acute and chronic oxidative stress models impaired intestinal barrier function and liver function. Chronic stress caused by D-galactose can result in severe redox dysregulation, while acute stress caused by paraquat can lead to inflammation and liver damage. Additionally, the components involved in the CAR pathway were expressed differently. Chronic or acute oxidative stress can reduce the diversity and composition of intestinal flora. In the PQ group, the richness of Mogibacterium and Denitratisoma improved, but in the D-gal group, the richness of Catenisphaera and Syntrophococcus increased.

**Discussion:**

Not only does this research deepen our understanding of the effects of acute and chronic oxidative stress on intestinal functions, but it also characterizes characteristic changes in the gut flora, potentially identifying novel therapeutic targets and opening new avenues for future research.

## 1 Introduction

An oxidative stress condition is defined as the accumulation of reactive oxygen species (ROS) along with a dysfunctional antioxidant system (Forman and Zhang, 2021), which also frequently occurs alongside inflammation and can exacerbate it. Although there is no precise definition, oxidative stress is generally classified as either acute or chronic based on its duration of action. Acute oxidative stress is involved in ischemic stroke, spinal cord injury, acute lung injury, and acute kidney injury, among others, while chronic oxidative stress plays a role in vascular dementia, nonalcoholic liver disease, chronic pancreatitis, and aging (Hajam et al., 2022). Although both result in damage to many intracellular components, particularly mitochondria and DNA (Li X. et al., 2022), there are differences in the mechanisms of acute versus chronic oxidative stress. For instance, in acute lung injury, the production of excess Nitric oxide (NO) induced by ROS causes bronchial arterioles to dilate, increases permeability, and directs blood flow into the lungs through bronchial-pulmonary vascular anastomotic branches, leading to pulmonary edema (Ward, 2010). On the other hand, during cerebral hypoperfusion, increased ROS in the vascular endothelium impairs NO signaling and reduces NO bioavailability (Liu and Zhang, 2012). In addition, excessive ROS in chronic pancreatitis primarily originates from the activation of cytochrome P450 enzymes, and antioxidant therapy has been found to be effective (Petrov, 2010). These studies indicate that acute and chronic oxidative stress have distinct mechanisms of action.

The intestine is a significant source and target of ROS (Feng et al., 2020) and can also induce liver lesions through the gut-liver axis. Through the portal vein system, there is a close anatomical and functional association between liver and intestine. Excessive accumulation of ROS can damage the tight junction proteins between intestinal epithelia, leading to intestinal mucosal epithelial cells becoming abnormally proliferative and differentiated (Liu et al., 2021). It can cause intestinal mucosal barrier dysfunction and alter the intestinal flora (Han et al., 2021). A substantial body of evidence from animal studies indicates that gut microbiota dysbiosis can lead to hepatic injury due to increased gut permeability, which in turn results in increased hepatic exposure to harmful substances (Krawczyk et al., 2018; Rainer et al., 2018). For instance, a significant number of patients with inflammatory bowel disease (IBD) also suffer from Nonalcoholic Fatty Liver Disease (NAFLD) (Younossi et al., 2016). The liver affects the state of intestinal oxidative stress by secreting bile and antibody components into the intestines, providing feedback to the intestines. Individuals with cholangitis have a significantly different intestinal flora than healthy individuals (Tilg et al., 2022). Oxidative stress has a crucial role to liver-gut axis interactions, particularly in intestinal injury. However, previous studies have generally described oxidative stress without distinguishing between acute and chronic forms. Therefore, few comparisons between these two types of oxidative stress have been reported.

Here we constructed two animal models of acute and chronic oxidative stress piglets. Paraquat was used to construct the acute oxidative stress models which is widely used in mammals (Xiang et al., 2022; Xiao et al., 2022). D-galactose, which was previously reported its used in rats and pig models (Sha et al., 2021; Sun et al., 2023), was used in chronic oxidative stress modeling. This study aimed to develop a model of acute and chronic oxidative stress in the porcine intestine and systematically examine its impact on the growth performance, the gut-liver axis and intestinal flora in weaned piglets.

## 2 Method

### 2.1 Animal and experimental design

A selection with 21 healthy weaned piglets (Duroc×Landrace×Large) was randomly separated into three groups: control (CON) group, D-galactose (D-gal) group, and paraquat (PQ) group, each with 7 replicates and 1 piglet per pen with comparable body weight (BW = 9.49 ± 0.26 kg). The basal diets were prepared with the NRC (2012) recommended nutrient requirements for piggies weighing 11 ~ 25 kg (Table 1). The experiment was divided into three phases. During the first phase, 5 days of adaptation was followed by feeding all piglets with the basal diet. In the second phase, the CON and PQ groups were fed the basal diet, while the D-gal group was fed the basal diet supplemented with 10 g/kg BW of D-galactose (Hubei Yuying Biotech. Company, Yichang, China) during a chronic stress period. The third phase was the acute stress period. In this phase, the PQ group received intraperitoneal injections of 8 mg/kg BW of PQ (Chengdu HuaXia Chemical Reagent, Chengdu, China) on the days 28, 30, and 32 (Xiang et al., 2022), while the CON group received equal volume of 0.9% sterile saline until they were slaughtered on day 33. The piglets were housed individually, provided with *ad libitum* feed and water throughout the experiment.

**Table 1:** Ingredients and nutrient levels of basal diets (air-dry basis, %).

Ingredients	Content	Nutrient level^†^	Content
Ripening corn	31.45	NE, kcal/kg	2,450
Ripening rice	25.60	EE	3.76
Flour	12.50	CP	17.32
Extruded soybean	6.40	*CF*	2.18
Fermented soybean meal	5.00	P	0.54
Ripening soybean meal	5.00	Ca	0.63
Fish meal	3.50	Total Lysine	1.39
Sucrose	2.50	Total Valine	1.15
Glucose	2.50	Total Threonine	0.95
Soybean oil	1.25	Total Tryptophan	0.29
50% Choline chloride	0.10	Total Methionine	0.65
CaHPO_3_	0.80		
Lysine · HCl	0.77		
Limestone	0.50		
NaCl	0.40		
L-Valine	0.38		
Methionine	0.38		
Threonine	0.36		
Premix^1^	0.19		
L-Tryptophan	0.12		
Total	100.00		

NE, net energy; EE, ether extract; CP, crude protein; CF, crude fiber. *Per kilogram of the diet, the premix offered the following: vitamin D, 3000 IU; vitamin A, 10,000 IU; vitamin E, 30 mg; vitamin B2, 10 mg; vitamin B6, 6 mg; vitamin B1 4 mg; vitamin K3, 4 mg; vitamin B12, 0.04 mg; nicotinic acid, 40 mg; pantothenic acid, 20 mg; folic acid, 2 mg; biotin, 0.2 mg; Fe (as ferrous sulfate), 100 mg; Zn (as zinc sulfate), 80 mg; Cu (as copper sulfate pentahydrate), 25 mg; Mn (as manganese sulfate), 25 mg; Co (as cobalt chloride), 0.5 mg; I (as Calcium iodate), 0.8 mg and Se (as sodium selenite), 0.35 mg. ^†^NRC models were used to compute net energy, and values for other nutrients were measured.

### 2.2 Sample collection

During the trial, the body weights of the piglets were recorded on days 1, 7, 14, 21, and 28. Daily feed intake and diarrhea were accurately documented. For each week, the average daily feed intake (ADFI), average daily gain (ADG), feed conversion ratio (FCR) and diarrhea rate (DR) were calculated. At the end of the experiment, blood was drawn from the anterior vena cava. After standing for 2 h, the blood was centrifuged for 10 min at 3,000 rpm at 4°C to extract the serum. For histological and pathological examination, 1 cm of liver, jejunum, and ileum tissues were extracted and instantly preserved in 3% glutaraldehyde solutions and 4% paraformaldehyde. For additional analysis, the liver, jejunum, ileum, and colon chyme were obtained, quick frozen in liquid nitrogen and then kept as low as −80°C.

### 2.3 Serum biochemical and physiological properties

A Cobas c 311 automated biochemical analyzer (Roche, Basel, Switzerland) was used to measure serum total protein (TP), immunoglobulin G (IgG), immunoglobulin M (IgM), alanine aminotransferase (ALT), aspartate aminotransferase (AST), and alkaline phosphatase (ALP). Lactate dehydrogenase (LDH), malondialdehyde (MDA), superoxide dismutase (SOD), total antioxidant capacity (T-AOC) and catalase (CAT) levels are measured with a colorimetric test kit (Beijing BoxBio Science & Tech. Co., Ltd., Beijing, China). and the process was completed in compliance with the instructions of the kit. The levels of glutathione (GSH), glutathione peroxidase (GSH-Px), oxidized glutathione (GSSG), immunoglobulin A (IgA), interleukin 1-β (IL-1β), interleukin-10 (IL-10), interleukin-12 (IL-12), tumor necrosis factor-α (TNF-α), interferon-γ (IFN-γ), intestinal fatty acid binding protein (iFABP), diamine oxidase (DAO), and adenosine triphosphate (ATP) were measured by ELISA (Jiangsu Meimian Industrial, Jiangsu, China), the detailed procedure is based on the kit protocol.

### 2.4 Tissue histomorphology

Hematoxylin and eosin (H&E) staining was performed on the jejunal and ileal sections after fixing with 4% paraformaldehyde. Villus height and crypt depth were assessed blindly by two separate investigators, same like in the previous study (He et al., 2022). The ileum and liver sections were pre-fixed with 3% glutaraldehyde for lead citrate and uranyl acetate staining. Certain lesions were observed using the JEM-1400 FLASH transmission electron microscope (JEOL, Tokyo, Japan), as reported (Hu et al., 2022).

### 2.5 Cell apoptosis

A terminal deoxynucleotidyl transferase dUTP nick end labeling (TUNEL) apoptosis assay kit (Beyotime Biotech, Shanghai, China) was used to detect apoptosis. Additionally, sections were examined with DM3000 fluorescence microscopy (Leica, Shanghai, China).

### 2.6 Immunohistochemical analysis

Immunohistochemical analysis was performed to examine the expression of Occludin, ZO-1 and Claudin 1 in the ileum and jejunum, as previously described (Xiang et al., 2022). Antibodies against the three proteins were used at the following dilution ratios: Claudin 1 (1: 500), Occludin (1: 1000), and ZO-1 (1: 800) (all made by Proteintech, Rosemont, IL, USA).

### 2.7 Real-time PCR analysis

The quantitative real-time PCR was performed as previously described (He et al., 2017). The primers were designed by Primer Express 5.0 software (Table 2) according to the conserved sequences of pig genes in the NCBI database and the principles of primer design. Total RNA was extracted with TRIzol (Beyotime, Shanghai, China) from the liver and jejunum tissue. The transcript levels of target genes were normalized to either GAPDH or β-actin expression. The relative level of mRNA was normalized to the CON group.

**Table 2:** Primers for quantitative reverse transcription-PCR.

Accession No.	Gene	Sequence primers (5′ → 3′)
NM_214407.1	GPX4	F: GATTCTGGCCTTCCCTTGC R: TCCCCTTGGGCTGGACTTT
NM_214201.1	GPX1	F: TGGGGAGATCCTGAATTG R: GATAAACTTGGGGTCGGT
NM_001190422.1	CuZnSOD	F: TGAAGGGAGAGAAGACAGTGTTAG R: TCTCCAACGTGCCTCTCTTG
NM_214127.2	MnSOD	F: GGACAAATCTGAGCCCTAACG R: CCTTGTTGAAACCGAGCC
XM_001926378.4	GCLM	F: CACAGCGAGGAGCTTCGAGA R: TGCGTGAGACACAGTACATTCC
XM_021098556.1	GCLC	F: GATCCTCCAGTTCCTGCACA R: GAGAGAGAACCAACCTCGTCG
NM_213948.1	IFN-γ	F: CAGGCCATTCAAAGGAGCAT R: GAGTTCACTGATGGCTTTGCG
NM_214041.1	IL-10	F: CGGCGCTGTCATCAATTTCTG R: CCCCTCTCTTGGAGCTTGCTA
NM_213993.1	IL-12	F: CAGGCCCAGGAATGTTCAAA R: CGTGGCTAGTTCAAGTGGTAAG
NM_214055.1	IL-1β	F: CCAATTCAGGGACCCTACCC R: GTTTTGGGTGCAGCACTTCAT
NM_001037996.1	CAR	F: GTGCCTGAACTGTCTCTGCT R: CCACATGCGCTCCATCTTCT
XM_003131409.5	CCRP	F: TGCCCTAGAATTTGCCCCTG R: GCAAAGACCTCGGACGTACA
XM_001927453.2	RXRα	F: CAAGTGCCTGGAACACCTCT R: ATGGAAGGTAACAGGGTGGC
NM_214366.1	PP2Ac	F: GGTGCCATGACCGGAATGTA R: GTGCTGGGTCAAACTGCAAG
NM_213973.1	HSP90	F: AAGACCGGACCCTCACGATA R: AGGCATACTGCTCGTCATCG
NM_213850.2	GSTA2	F: CTACTACGTGGAAGAGCTGGAC R: GCCCTGCCCACTTTATGAAGAC
NM_214389.2	GSTA1	F: AGGACACCCAGGACCAATCTT R: CTCAGGTACATTCCGGGAGAAG
NM_001159614.1	CYP1A2	F: TTTGTGGAGACCGCCTCATC R: GCTTGAATAGGGCGCTTGTG
NM_214423.1	CYP3A29	F: CCTGAAATTAACCACGCAAGGGCT R: TCTGGGATGCAGCTTTCTTGACCA
NM_214413.1	CYP2B22	F: GGGAACGTTGGAAGACCCTT R: CGGGATCTCTGTAGGCGAAG
NM_001206359.1	GAPDH	F: AAGGAGTAAGAGCCCCTGGA R: TCTGGGATGGAAACTGGAA
XM_003124280.3	β-actin	F: CTGCGGCATCCACGAAACT R: AGGGCCGTGATCTCCTTCTG

### 2.8 Gut microbiota profiles

Total microbial DNA was extracted from colon contents samples utilizing the TGuide S96 Magnetic Bead Method Soil/Fecal Genomic DNA Extraction Kit (Tiangen, Beijing, China). Using the primers for the V3-V4 region of the bacterial DNA gene (338F: 5’-ACTCCTACGGGGAGGCGCAGCA-3′ and 806R: 5’-GGACTAC HVGGGTWTCTAAT-3′), 5–50 ng of DNA was extracted and amplified by PCR. The amplified products were homogenized and purified, the libraries were quality-checked by the Qsep-400 method, and the libraries were sequenced using the Illumina Novaseq 6000 platform. High-quality sequences were obtained by first quality filtering the raw sequencing data using Trimmomatic (version 0.33), then identifying and removing primer sequences using Cutadapt (version 1.9.1), splicing bipartite reads using USEARCH (version 10), and lastly removing chimeras using UCHIME (version 8.1). OTUs were classified by clustering/de-noising high-quality sequences, and their species categorization was achieved by analyzing the sequence composition of the OTUs. The BMKCloud^1^ platform was used for subsequent bioinformatics research on microbial diversity and differentiation. Raw sequence data reported in this paper have been deposited (CRA015743) in the Genome Sequence Archive in the BIG Data Center, Chinese Academy of Sciences^2^ (Members, 2018).

### 2.9 Statistical analysis

The data were analyzed using SPSS 20.0 (IBM-SPSS, Chicago, USA) and graphics were processed with GraphPad Prism 8.0 (GraphPad Software, San Diego, USA). Data were analyzed statistically using a *t*-test or one-way ANOVA and Duncan’s multiple comparisons test. The data are shown as mean ± standard error, with *p* < 0.05 suggesting a significant difference between groups.

## 3 Results

### 3.1 Effect of acute and chronic oxidative stress on intestinal morphology and growth performance in weaned piglets

**Figure 1:**
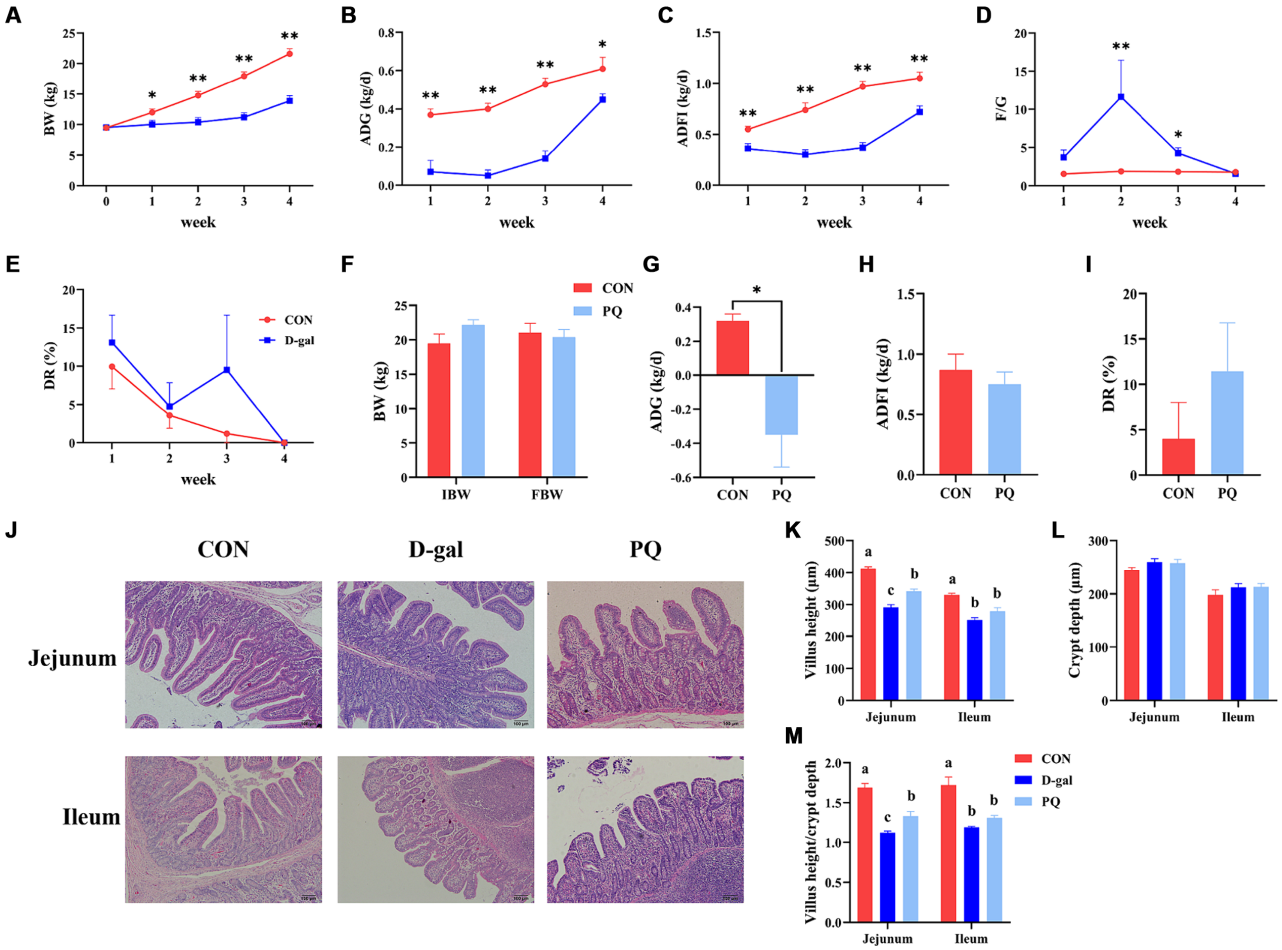
Effect of acute and chronic oxidative stress on growth performance and intestinal morphology in weaned piglets. **(A–E)** The BW, ADG, ADFI, F/G, and DR per week for CON group and D-gal group. **(G–I)** The ADG, ADFI, and DR for CON group and PQ group. **(J)** Representative H&E staining of jejunal and ileal tissue for weaned piglets (×100; Scale bars = 100 μm). **(K–M)** The villus height, crypt depth and Villus height to crypt depth ratio of ileum and jejunum in weaned piglets. A significant difference (*p* < 0.05) is indicated by different tiny letter superscripts, whereas no significant difference (*p* > 0.05) is indicated by the same or no letter superscripts. The values are given as Mean ± SE, *n* = 7. CON group, control group; D-gal group, D-galactose induced chronic oxidative stress group; PQ group, paraquat induced acute oxidative stress group; BW, body weight; ADG, average daily gain; ADFI, average daily feed intake; F/G, feed/gain; DR, diarrhea rate.

In comparison to the CON group, the results demonstrated that D-galactose treatment dramatically decreased BW, ADG, and ADFI during the course of the four weeks (Figures 1A–C). Diarrhea rates had no discernible difference in the two groups and both decreased to 0 at 4 weeks as piglets adapted to the environment (Figure 1E). However, at the third week, the D-gal group observed an 8.3% greater rate of diarrhea against the CON group. Furthermore, compared to the CON group, the F/G ratio in the D-gal group exhibited a significant increase in both the second and third weeks (Figure 1D). The ADG was significantly decreased after the paraquat challenge (Figure 1G). However, there were no appreciable variations in rate of diarrhea, BW or ADFI (Figures 1F,H,I).

Oxidative stress induces damage to the intestinal morphology, which is linked to disruptions in the gut epithelial barrier. As shown in our work, PQ and D-galactose treatment resulted in jejunal and ileal morphological damage in weaned piglets (Figure 1J). While the two treatments did not significantly alter the crypt depth (Figure 1L), both PQ and D-galactose challenges, compared to the CON group, caused a drop in the villus height and the villus height to crypt depth ratio (V/C). Additionally, a shorter villi and V/C ratio were observed in the D-gal group (Figures 1K,M). The results suggest that both D-galactose and paraquat have a negative impact on the jejunal and ileal morphology, as well as on the growth performance. However, D-galactose induced chronic oxidative stress caused a more severe impairment of growth potential and morphology.

### 3.2 Effect of acute and chronic oxidative stress on intestinal permeability in weaned piglets

**Figure 2:**
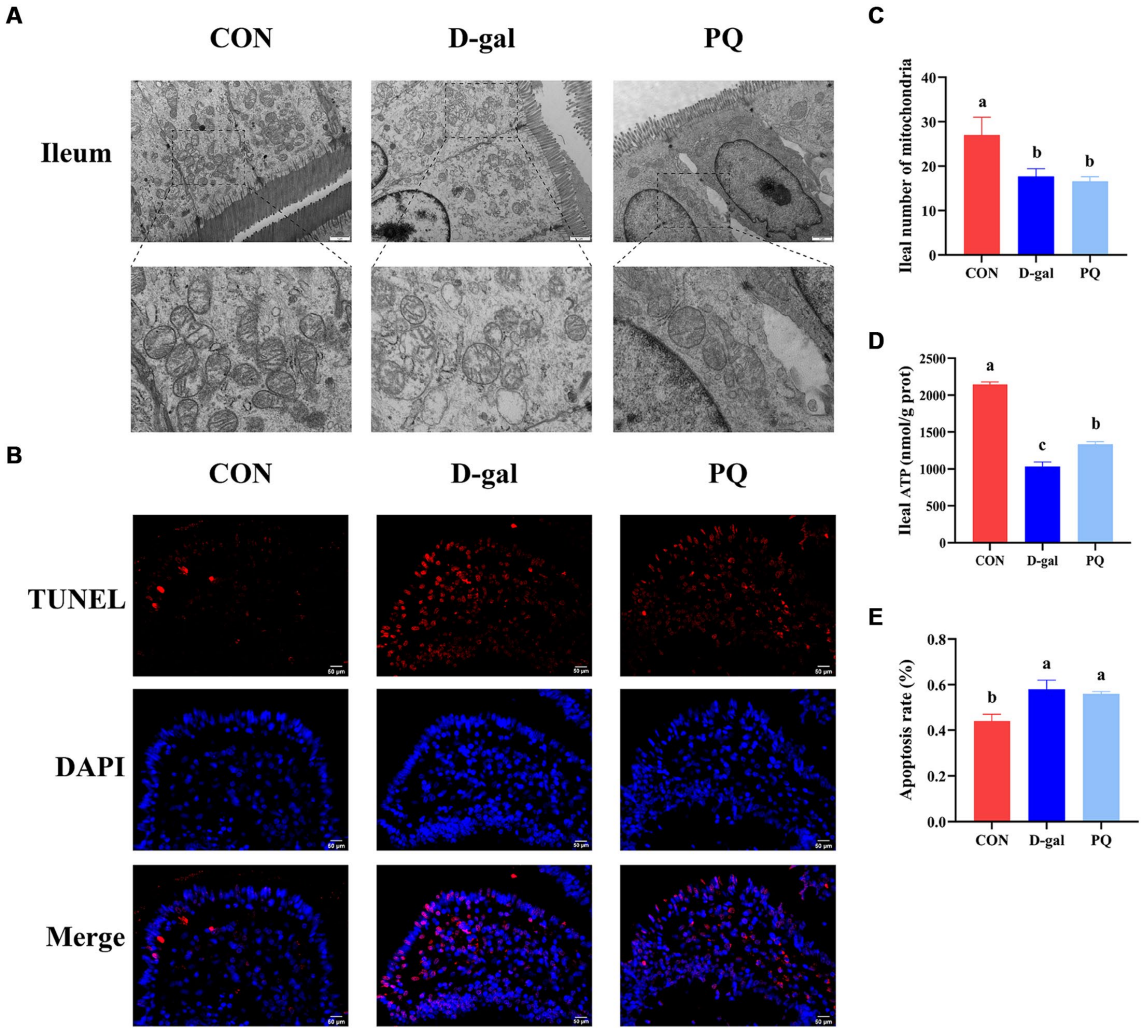
Effect of acute and chronic oxidative stress on intestinal epithelial cell in weaned piglets. **(A)** Epithelial cells ultrastructure in the ileum for different group (×15,000; Scale bars = 1 μm). **(B)** Representative TUNEL staining of ileal tissue (Scale bars = 50 μm; ×400). **(C)** Number of mitochondria in ileal epithelial cells. **(D)** ATP content in ileal epithelial cells. **(E)** The apoptosis rate quantitation in the ileum. A significant difference (*p* < 0.05) is indicated by different tiny letter superscripts, whereas no significant difference (*p* > 0.05) is indicated by the same or no letter superscripts. The values are given as Mean ± SE, *n* = 7. CON group, control group; D-gal group, D-galactose induced chronic oxidative stress group; PQ group, paraquat induced acute oxidative stress group.

We evaluated the structure and protein abundances of the tight junction, which are crucial for preserving the integrity and impermeability of the intestinal mucosal barrier. When stimulated by PQ or D-galactose, the intestinal microvilli of piglets were found to be short, thick, loosely organized, and physiologically uneven, as shown in the transmission electron microscope image (Figure 2A). Meanwhile, the group treated with PQ and D-galactose exhibited heterogeneous and fewer mitochondria compared to the control group (CON) (Figure 2C), with reduced and broken cristae. Additionally, the PQ and D-galactose group significantly reduced the number of mitochondria in the ileum and ATP levels (Figure 2D). Weaned piglets treated with PQ or D-galactose showed increased intestinal epithelial cell apoptosis as detected by immunofluorescence (Figures 2B,E).

**Figure 3:**
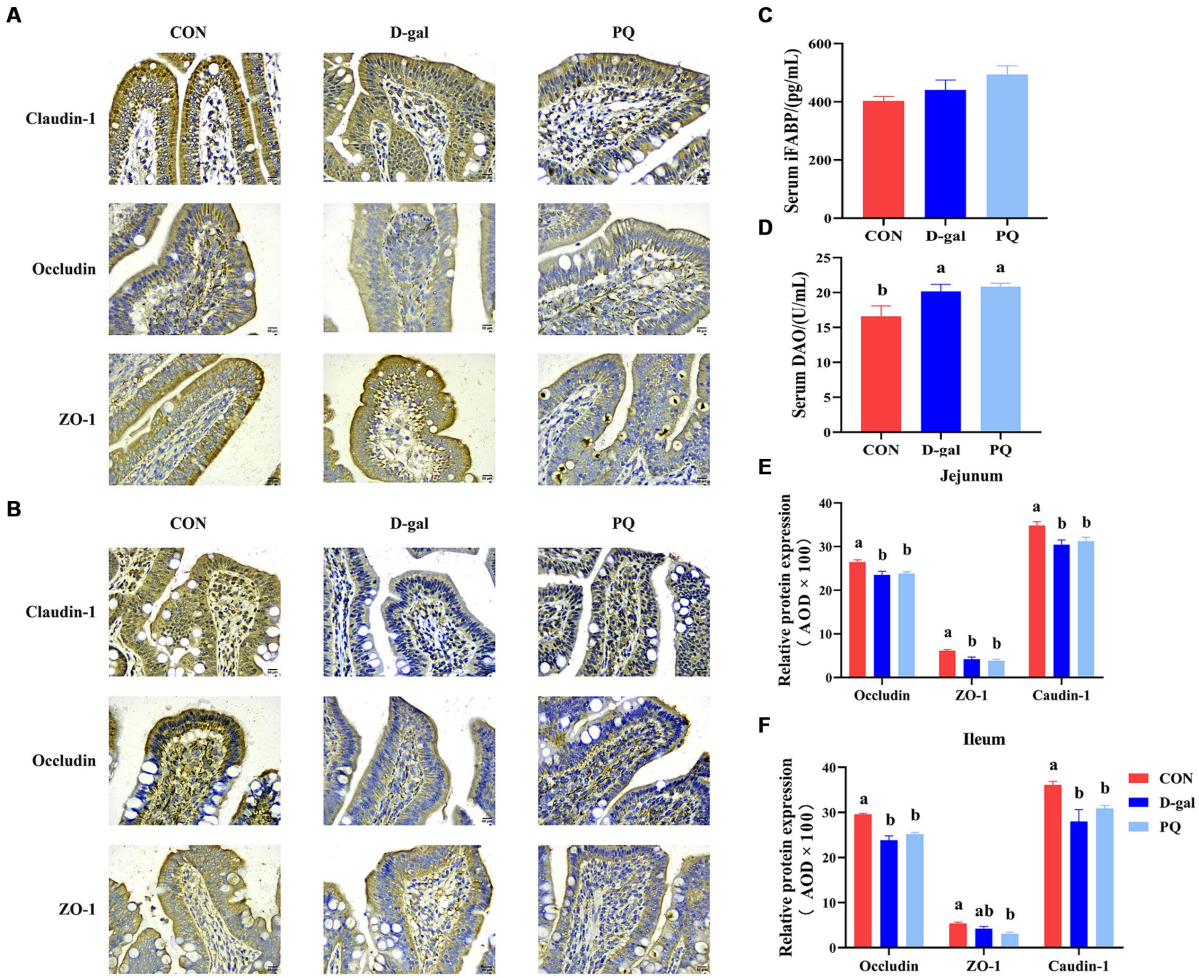
Effect of acute and chronic oxidative stress on intestinal barrier function in weaned piglets. Immunohistochemical staining of Claudin-1, Occludin and ZO-1 of jejunum **(A)** and ileum **(B)** (Scale bars = 50 μm; ×400). **(C,D)** Level of serum DAO and iFABP. **(E,F)** The relative expression of Claudin-1, Occludin and ZO-1 in the jejunum and ileum. A significant difference (*p* < 0.05) is indicated by different tiny letter superscripts, whereas no significant difference (*p* > 0.05) is indicated by the same or no letter superscripts. The values are given as Mean ± SE, *n* = 7. CON group, control group; D-gal group, D-galactose induced chronic oxidative stress group; PQ group, paraquat induced acute oxidative stress group; AOD, average optical density.

The effect of PQ and D-galactose on intestinal barrier function was further investigated by examining the localization and expression of intestinal tight junctions, such as Claudin-1, ZO-1, and Occludin protein. The findings (Figures 3A,B,E,F) demonstrated that the expression of Claudin-1, ZO-1, and Occludin protein in the jejunum and ileum of weaned piglets was significantly inhibited by challenging with PQ or D-galactose. Additionally, intestinal permeability was altered (Figures 3C,D), and the DAO content was markedly higher than that of the CON group. As hypothesized, the challenge of PQ and D-galactose could increase the intestinal permeability of weaned piglets.

### 3.3 Effect of acute and chronic oxidative stress on the gut-liver axis in weaned piglets

**Figure 4:**
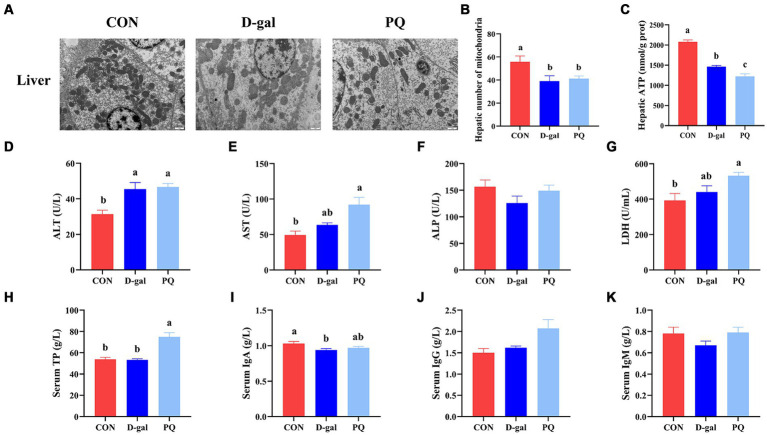
Effect of acute and chronic oxidative stress on the gut-liver axis in weaned piglets. **(A)** Liver cells ultrastructure in the liver for different group (×15,000; Scale bars = 1 μm). **(B)** Number of mitochondria in liver cells. **(C)** Level of ATP in liver cells. **(D–K)** The level of ALT, AST, ALP, LDH, TP, IgA, IgG, and IgM in serum. A significant difference (*p* < 0.05) is indicated by different tiny letter superscripts, whereas no significant difference (*p* > 0.05) is indicated by the same or no letter superscripts. The values are given as Mean ± SE, *n* = 7. CON group, control group; D-gal group, D-galactose induced chronic oxidative stress group; PQ group, paraquat induced acute oxidative stress group.

TEM confirmed the morphology and quantity of hepatocyte mitochondria (Figure 4A). The PQ group had fewer and more heterogeneous mitochondria than the CON group, with reduced and broken cristae (Figure 4B). Additionally, the hepatic ATP level was significantly decreased (Figure 4C). The PQ group had considerably greater levels of ALT (Figure 4D), AST (Figure 4E), LDH (Figure 4G), and serum TP (Figure 4H) compared with the CON group. However, PQ had little impact on the levels of ALP (Figure 4F), serum IgA (Figure 4I), serum IgG (Figure 4J), and serum IgM (Figure 4K). The D-galactose challenge resulted in similar changes, although not all differences were statistically significant (Figures 4A–K). It is noteworthy that the PQ group had higher levels of AST than the D-gal group. Taken together, our findings indicate that both PQ and D-galactose challenge can damage hepatocytes and their redox system and the PQ group suffered more severe hepatic impairment.

### 3.4 Effect of acute and chronic oxidative stress on antioxidant capacity and inflammatory cytokines in weaned piglets

**Figure 5:**
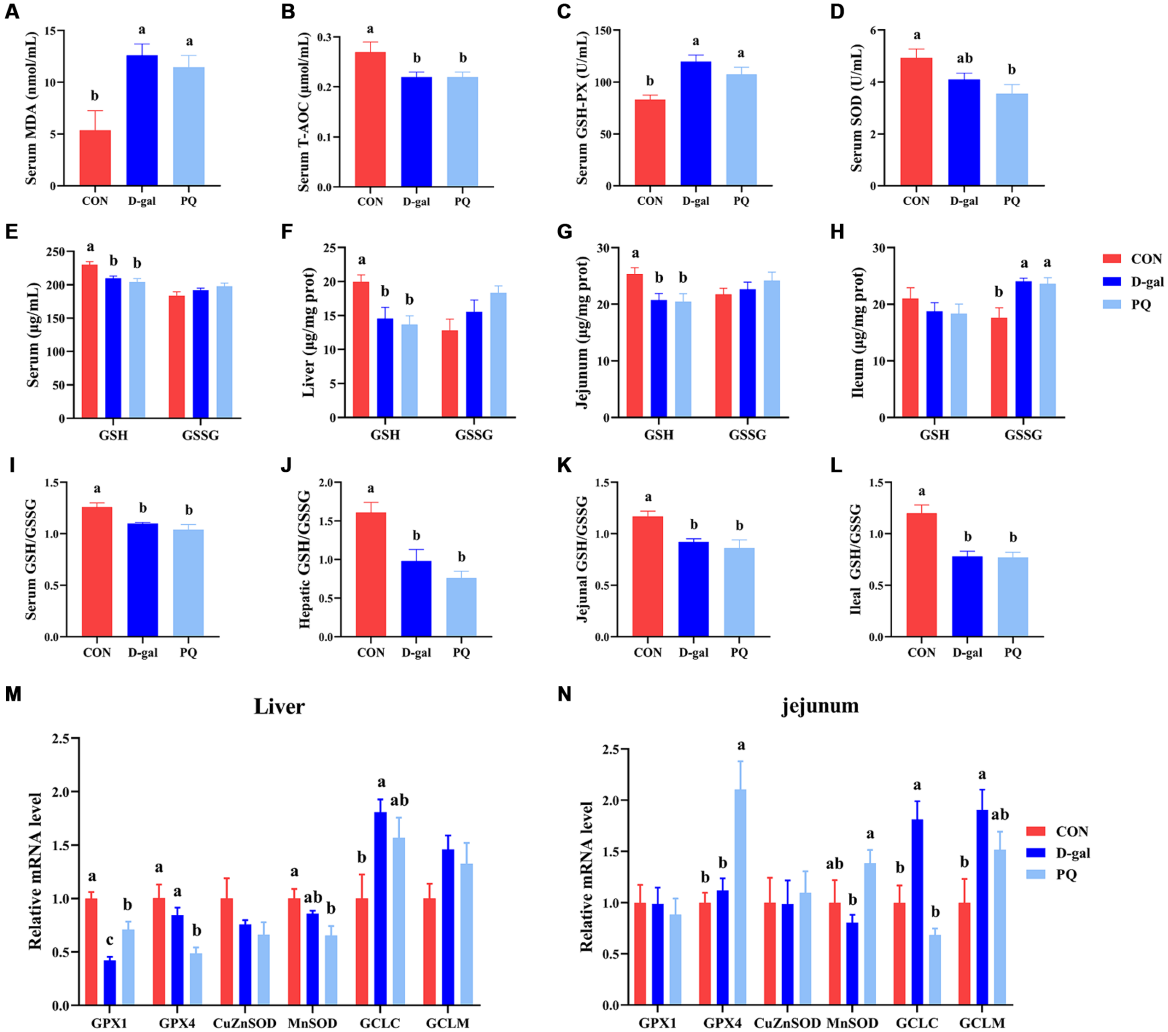
Effect of acute and chronic oxidative stress on antioxidant capacity in weaned piglets. **(A–D)** The level of MDA, T-AOC, GSH-PX and SOD in serum. The level of GSH and GSSG in serum **(E)**, liver **(F)**, jejunum **(G)**, and ileum **(H)**. The GSH/GSSG ratio in serum **(I)**, liver **(J)**, jejunum **(K)**, and ileum **(L)**. The relative express of GPX1, GPX4, CuZnSOD, MnSOD, GCLC, and GCLM mRNA in liver **(M)** and jejunum **(N)**. A significant difference (*p* < 0.05) is indicated by different tiny letter superscripts, whereas no significant difference (*p* > 0.05) is indicated by the same or no letter superscripts. The values are given as Mean ± SE, *n* = 7. CON group, control group; D-gal group, D-galactose induced chronic oxidative stress group; PQ group, paraquat induced acute oxidative stress group; MDA, malondialdehyde; T-AOC, total antioxidant capacity; GSH-Px, glutathione peroxidase; SOD, superoxide dismutase; GSH, glutathione; GSSG, oxidized glutathione.

To better understand the impact of acute and chronic oxidative stress on the redox system, we examined serological and intestinal antioxidant-related enzymes. The PQ and D-galactose challenge both significantly raised the serum MDA content (Figure 5A) and GSH-PX activity (Figure 5C) compared to the CON group, but a decline in serum T-AOC activity (Figure 5B) and serum SOD (Figure 5D). The ratio of GSH/GSSG in serum, liver, jejunum, and ileum were significantly decreased by PQ and D-galactose challenge (Figures 5E–L). This is due to the fact that the GSH/GSSG ratio is calculated from the previous GSH and GSSH values. Consistent with the results above, our results suggest a decreasing trend in the CuZnSOD and MnSOD mRNA expression while the expressions of GCLC and GCLM tend to increase after PQ or D-galactose challenge. (Figures 5M,N). And this trend was evident in the D-gal group. Our findings demonstrate that PQ and D-galactose challenge disrupt redox balance and impairs antioxidant capacity in weaned piglets.

**Figure 6:**
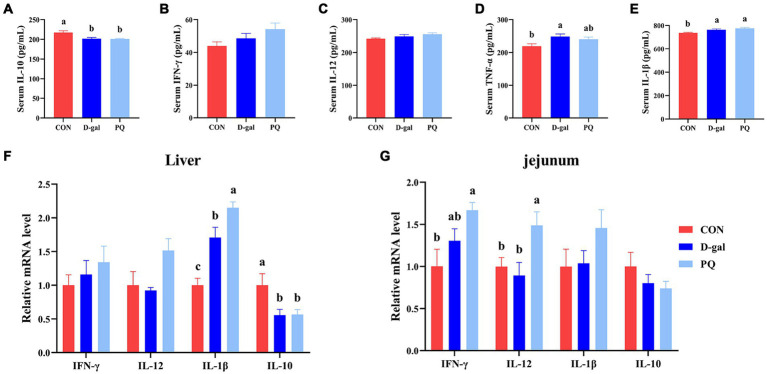
Effect of acute and chronic oxidative stress on inflammatory cytokines in weaned piglets. **(A–E)** The level of IL-10, IFN-γ, IL-12, TNF-α, and IL-1β in serum. The relative express of IL-10, IFN-γ, IL-12, TNF-α, and IL-1β mRNA in liver **(F)** and jejunum **(G)**. A significant difference (*p* < 0.05) is indicated by different tiny letter superscripts, whereas no significant difference (*p* > 0.05) is indicated by the same or no letter superscripts. The values are given as Mean ± SE, *n* = 7. CON group, control group; D-gal group, D-galactose induced chronic oxidative stress group; PQ group, paraquat induced acute oxidative stress group.

Since oxidative stress typically triggers an inflammatory response, we further assessed the inflammatory level of weaned piglets. PQ or D-galactose exposure elevated the level of type 1 cytokines in serum, such as IL-1β (Figure 6E) and TNF-α (Figure 6D), which promote type I inflammation. There is no significant difference in IFN-γ and IL-12 (Figures 6B,C). However, PQ and D-galactose decreased the level of IL-10 in serum (Figure 6A), which regulates immunity. Similar results were found in the liver (Figure 6F) and jejunum (Figure 6G) tissues, where PQ or D-galactose increased IL-12 and IFN-γ levels and decreased IL-10 levels. Notably, the PQ group had larger levels of IL-1β, IFN-γ, and IL-12 than the D-gal group did. Overall, the results suggest that PQ and D-galactose induce type 1 proinflammatory cytokines and cause an imbalance in immunity.

### 3.5 Effect of acute and chronic oxidative stress on CAR pathway in weaned piglets

**Figure 7:**
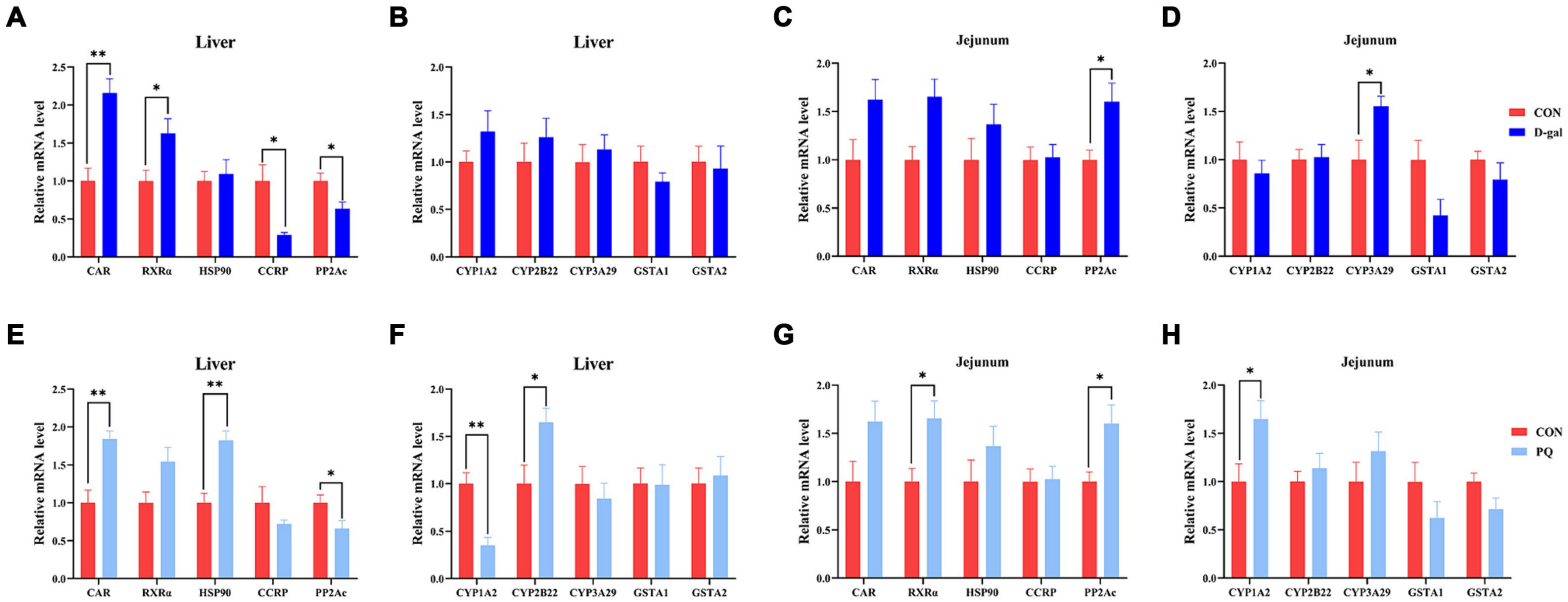
Effect of acute and chronic oxidative stress on CAR pathway in weaned piglets. **(A–D)** The relative mRNA expression of CAR, RXRα, HSP0, CCRP, PP2Ac, CYP1A2, CYP2B22, CYP3A29, GSTA1, and GSTA2 in liver and jejunum for CON group and D-gal group. **(E–H)** T The relative mRNA expression of CAR, RXRα, HSP0, CCRP, PP2Ac, CYP1A2, CYP2B22, CYP3A29, GSTA1, and GSTA2 in liver and jejunum for CON group and PQ group. A significant difference (*p* < 0.05) is indicated by different tiny letter superscripts, whereas no significant difference (*p* > 0.05) is indicated by the same or no letter superscripts. The values are given as Mean ± SE, *n* = 7. CON group, control group; D-gal group, D-galactose induced chronic oxidative stress group; PQ group, paraquat induced acute oxidative stress group.

To fend off oxidative stress brought on by both internal and external stressors, the Constitutive Androstane Receptor (CAR) has the ability to control the production of antioxidant genes like SOD, CAT, and CYP450. In this study, we aimed to determine the potential mechanism underlying the effects of PQ and D-galactose on weaned piglets by examining key targets of the CAR pathway. Compared to the CON group, the PQ group showed an increase in mRNA expression of CAR, HSP90, and CYP2B22 (Figures 7E,F), while also showing a decrease in mRNA expression of PP2Ac and CYP1A2 (Figures 7E,F) in the liver. However, the jejunum of the PQ group showed an increase in expression of RXRα, PP2Ac, and CYP1A2 (Figures 7G,H). D-galactose caused the hepatic CCRP and PP2Ac expression to be downregulated, while the expression of PP2Ac and CYP3A29 in the jejunum was upregulated (Figures 7A,C,D). The expression of PP2Ac and CYP3A29 showed no significant difference in liver though (Figure 7B). These findings imply that the CAR signaling pathway may play a different role in liver and gut antioxidant function impairment following stimulation with PQ or D-galactose.

### 3.6 Effect of acute and chronic oxidative stress on intestinal flora in weaned piglets

**Figure 8:**
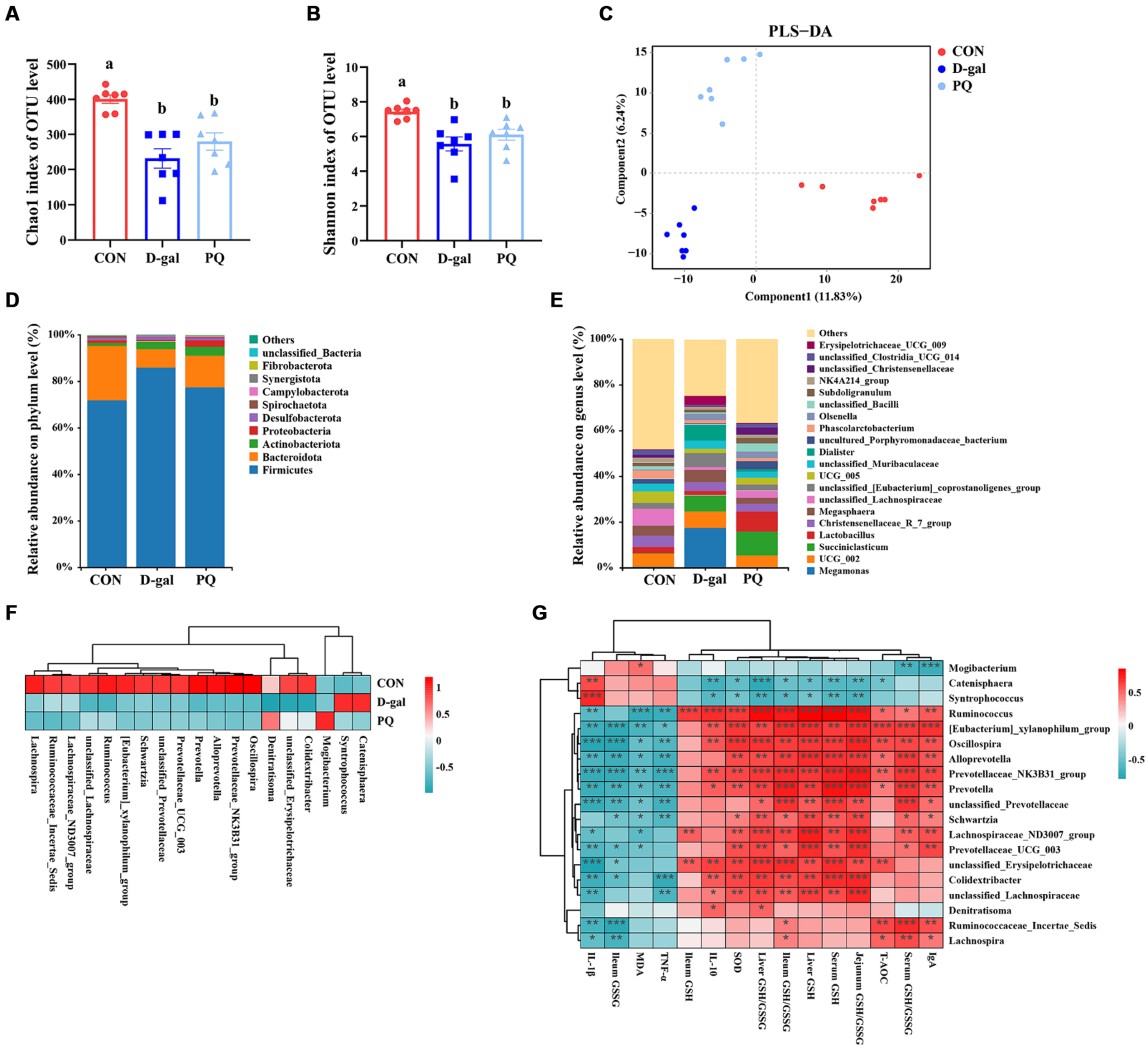
Effect of acute and chronic oxidative stress on intestinal flora in weaned piglets. **(A,B)** The Chao 1 index and Shannon index for different group. **(C)** PLS-DA scores plot among the CON group, PQ group, and D-gal group. **(D)** Differences in richness of intestinal flora at the phylum level. **(E)** Differences in richness of intestinal flora at the genus level. **(F)** Heatmap showing the differential richness of intestinal flora among the CON group, PQ group, and D-gal group. **(G)** Correlation analysis of differential flora with indicators of oxidative stress and inflammation. A significant difference (*p* < 0.05) is indicated by different tiny letter superscripts, whereas no significant difference (*p* > 0.05) is indicated by the same or no letter superscripts. The values are given as Mean ± SE, *n* = 7. CON group, control group; D-gal group, D-galactose induced chronic oxidative stress group; PQ group, paraquat induced acute oxidative stress group.

The delicate balance of the intestinal flora is essential for preserving intestinal immunity and overall body homeostasis. Many studies have shown a direct relationship between oxidative stress with intestinal flora. The total amount of intestinal flora was estimated to be 2.5 million tons, with 1.5 million bacteria contributing to the antioxidant effect. To identify the bacteria responsible for the antioxidant function, the V3 + V4 regions of 16S rRNA genes were sequenced in order to conduct a microbiota analysis. An average of 76,022 clean reads were clustered after low-quality sequences were eliminated. Based on 97% similarity level, the high-quality sequences were assigned to operational taxonomic units (OTUs). Community richness was assessed using the Chao 1 index, while the Shannon index was chosen as a composite measure of community diversity. Compared to the CON group, both PQ and D-galactose considerably reduced the Chao 1 and Shannon indexes (Figures 8A,B). We assessed β-diversity using Principal Component Analysis (PCA) in order to better understand the variations in microbial composition between the groups. The results indicate that there were variations in the microbial community structure among the three groups (Figure 8C).

The microbial composition of the group differed at the phylum and genus levels. Both PQ and D-galactose decreased the ratio of *Bacteroidota* while improving the relative richness of *Firmicutes* and *Actinobacteriota* at the phylum level (Figure 8D). At the genus level, the flora composition differed significantly among the three groups (Figure 8E), which is in line with the previous discoveries. We examined the relative richness and found that all 19 genera exhibited significant differences in richness between each pair of the three groups (Figure 8F). *Mogibacterium* and *Denitratisoma* increased in the PQ group, while *Catenisphaera* and *Syntrophococcus* increased in the D-galactose group. *Alloprevotella, Colidextribacter, Ruminococcaceae_Incertae_Sedis, Lachnospira, Lachnospiraceae_ND3007_group, Oscillospira, Prevotella, Prevotella Prevotellaceae_NK3B31_group, Prevotellaceae_UCG_003, Ruminococcus, Schwartzia, [Eubacterium]_xylanophilum_group, unclassified_ Erysipelotrichaceae, unclassified_Lachnospiraceae,* and *unclassified_Prevotellaceae* were decreased in PQ and D-galactose group. We analyzed the association of these groups with the PQ and D-galactose group. In this study, we analyzed the association between these bacteria and oxidative reaction components and inflammatory cytokines (Figure 8G). These findings point to a possible connection between these bacteria and oxidative stress and inflammation. Our findings indicate that a high richness of *Catenisphaera* and *Syntrophococcus,* which increased in the D-galactose group, was associated with lower levels of T-AOC, GSH, ileum GSH/GSSG, SOD and IL-10, and higher level of IL-1β. Additionally, we observed that an increase in the richness of *Mogibacterium* in the PQ group corresponded to lower levels of IgA and serum GSH/GSSG, and higher levels of MDA. On the other hand, *Denitratisoma* tended to increase IL-10 and liver GSH/GSSG. The remaining 15 genera showed opposite effects. These findings suggest that PQ and D-galactose treatment can reduce the diversity and alter the richness of gut flora, leading to imbalances in oxidative and antioxidant processes and inflammatory reactions.

## 4 Discussion

The gut-liver axis maintains a dynamic balance through the interplay of the intestinal barrier, intestinal flora, and the biological functions of the liver (Ran et al., 2022). The multiple-hit hypothesis proposes that a combination of mechanical, chemical, immunologic, and biologic factors affect the intestinal mucosa. Once the intestinal mucosal barrier is disrupted, intestinal bacteria and their products migrate to the liver, causing a series of immune injuries and inflammatory responses that ultimately lead to liver injury. The intestine is susceptible to damage from ROS due to its exposure to dietary and environmental changes (Hu et al., 2022), as well as its abundance of intrinsically colonized immune cells and intestinal flora, which can produce ROS (Upadhaya and Kim, 2021). Uncontrolled ROS production can directly damage biomolecules and act as a second messenger to activate downstream inflammatory or cell death signaling pathways. Chronic oxidative stress can cause severe redox dysregulation, while acute oxidative stress can easily lead to inflammation and tissue damage. In our study, we used paraquat to generate a model of acute oxidative stress in weaned piglets and utilized D-galactose to create a model of chronic oxidative stress in piglets (Umbayev et al., 2020; Xiao et al., 2022). D-galactose is known to be metabolized to galactitol, which builds up in cells and produces an excess of ROS, causing chronic oxidative stress. The intraperitoneal injection of paraquat is absorbed by the intestinal mesentery and utilizes molecular oxygen within the bloodstream to generate excess ROS, leading to acute oxidative stress. Our data show that piglets given D-galactose displayed lower BW, ADG, and ADFI over a four-week period. Following the paraquat challenge, the ADG was reduced, but no notable variations were observed in BW, ADFI, or diarrhea rate. This suggested that D-galactose induced chronic oxidative stress may cause a more severe impairment of growth potential. These results are consistent with previous reports (Han et al., 2021; Xiang et al., 2022).

The intestine has two primary functions: selective absorption of nutrients and resistance to pathogens and toxins. These functions rely heavily on the intestinal barrier integrity. This study found that acute and chronic oxidative stress decreased the intestinal villus height and V/C ratio in piglets. Higher villi and shallower crypts suggest greater intestinal digestion and absorption, as well as faster cell maturation rates (Ou et al., 2020). This indicates that acute and chronic oxidative stress may impair intestinal absorption function. Oxidative stress damages the tight junctions, microvillus structure, and mitochondrial morphology of ileal epithelial cells. It also increases apoptosis of intestinal epithelial cells and decreases the expression of jejunal tight junction proteins, including Claudin-1, ZO-1, and Occludin. These proteins are critical components of the tight junction structure, which is essential for preserving intestinal permeability (Jiang et al., 2021). In summary, both acute and chronic oxidative stress can cause structural and functional damage to the intestinal tract of weaned piglets. This damage can lead to impaired intestinal barrier function, ultimately affecting piglet growth potential.

Antioxidants help the body defend against the detrimental effects of free radicals, which are continuously produced. Enzymatic and non-enzymatic systems that are comprised of antioxidants are referred to as the antioxidant defense system (Mendonca et al., 2022). GSH-Px, SOD, CAT, and GSH reductase are the main enzymatic antioxidants. Copper, zinc, and selenium are examples of nonenzymatic antioxidant systems that are essential for preventing oxidative stress. Our study found that both acute and chronic oxidative stress can lead to a rise in GSH-Px levels and a reduction in SOD levels in weaned piglets. This, in turn, causes a decrease in the GSH to GSSG ratio and T-AOC of antioxidant indexes and an increase in MDA of oxidized metabolites. The hepatic and jejunal CuZnSOD and MnSOD mRNA expression were reduced after acute and chronic oxidative stress, which coincided with a decrease in serum SOD levels. Glutamate cysteine ligase (GCL) is a rate-limiting enzyme of the cellular antioxidant GSH synthesis. Chronic oxidative stress induced by D-gal upregulated the expression of hepatic and jejunal GCLC and GCLM mRNAs, which are the catalytic and regulatory component of GCL (Lu, 2009). Overall, the antioxidant system is generally decompensated in the D-galactose induced chronic oxidative stress model.

Redox dysregulation in the intestine impairs liver function via the gut-liver axis. Particular markers of liver injury include serum AST, ALP, and ALT. Research has demonstrated that oxidative stress raises AST and ALT levels in the liver and serum in piglets (Zeng et al., 2021). When the liver is injured, the circulatory system receives a rise in AST and ALT concentrations in the serum. The study found that acute and chronic oxidative stress significantly impaired the number and function of mitochondria in hepatocytes. Additionally, serum ALT and AST levels were elevated, while ALP levels showed no statistically significant difference. Liver injury was observed in weaned piglets in both acute and chronic oxidative stress situations. However, statistically significant elevations in LDH and TP levels were observed only in the PQ-induced acute stress model. The heavier diarrhea in the acute model may have caused a short-term decrease in blood volume, which is consistent with the weight loss observed in the acutely stressed piglet group. Additionally, oxidative stress is connected to inflammatory reactions (Tian et al., 2023). Inflammatory processes could be induced by oxidative stress injury, and pro-inflammatory factors increase the production of ROS and RNS (Zong et al., 2023). The results showed that both acute and chronic oxidative stress can induce inflammation. During acute oxidative stress, the pro-inflammatory factors IL-1β and IFN-γ were upregulated, while the expression of the anti-inflammatory factor IL-10 was inhibited. Chronic oxidative stress additionally led to increased expression of TNF-α. Besides, the PQ group exhibited greater amounts of IL-1β, IL-12, and IFN-γ in contrast to the D-gal group. Both the liver and the jejunum showed a similar trend in inflammatory cytokine levels. The results indicate that acute and chronic oxidative stress cause local destruction of biomolecules and cellular structures, as well as changes in some physiological indicators in the intestine. Furthermore, they cause liver injury and systemic inflammation through the gut-liver axis. Combined with the previous results, it is evident that acute oxidative stress increases the risk of inflammation and liver damage, while chronic oxidative stress causes more severe antioxidant malfunction. This suggests that the molecular mechanisms involved in acute and chronic oxidative stress may differ.

We examined the expression of the CAR signaling pathway to better understand this mechanism. In a physical state, cytoplasmic CAR retention protein (CCRP) binds to HSP70/90 to hold CAR in the cytoplasm, creating the CAR-CCRP-HSP70/90 complex. CAR is translocated to the nucleus after being dephosphorylated by protein phosphatase 2A (PP2A). After that, it forms a heterodimer by binding to RXRα. This heterodimer modifies the activity of downstream target genes to regulate immunological and metabolic functions (Kobayashi et al., 2003). The results show that PQ or D-galactose treatment activated the CAR signaling pathway in both the jejunum and the liver tissues. The mRNA expression of CAR, RXRα, and HSP90 tended to increase, while the expression of PP2Ac increased in the jejunum tissues and decreased in the liver tissues, resulting in differences in the expression of downstream target genes of CAR. This is in line with the results of previous report (Tang et al., 2024), which found that oxidative stress induced by phosphorylates CAR, prevents the CAR-CCRP-HSP90 complex from forming, promotes CAR translocate to nucleus, and binds with RXRa, all of which control downstream target gene expression and hasten CAR activation. In summary, both PQ and D-galactose treatments can activate the CAR signaling pathway, leading to oxidative stress. However, there are differences, and even opposite trends, in the expression of CAR signaling pathway components between acute and chronic oxidative stress, as well as between the intestine and liver. These findings demonstrate differences in the expression of molecules involved in the CAR pathway under acute and chronic oxidative stress. Further studies may be necessary to explore additional mechanistic differences.

The intestinal tract possesses natural properties such as anaerobic, rich nutrition, appropriate temperature and PH, making it an excellent environment for microbial survival and hosting a large number of microorganisms (Zhou et al., 2020). These microorganisms form a complex biological system, also known as intestinal flora, whose diversity exerts a significant influence on the development of the disease (Xing et al., 2024).It is susceptible to attack by oxygen radicals under oxidative stress due to its active energy metabolism (Sebastián Domingo and Sánchez Sánchez, 2018). The relationship between gut flora and the host has drawn more and more attention from researchers in recent years. According to our study, the intestinal flora in weaned piglets significantly declined in the diversity and composition after PQ or D-galactose challenge. This suggests that the balance of intestinal flora can be disrupted by both acute and chronic oxidative stress. We also saw notable alterations in the microbial community structure. The study found that the PQ group experienced an enrichment of *Mogibacterium* and *Denitratisoma*, while the D-galactose intervention group experienced an enrichment of *Catenisphaera* and *Syntrophococcus.* Both intervention groups showed a downgrade of *Alloprevotella, Colidextribacter, Ruminococcaceae_Incertae_Sedis, Lachnospira, Lachnospiraceae_ND3007_group, Oscillospira, Prevotella, Prevotellaceae_NK3B31_group, Prevotellaceae_UCG_003, Ruminococcus, Schwartzia, [Eubacterium]_xylanophilum_group, unclassified_Erysipelotrichaceae, unclassified_Lachnospiraceae,* and *unclassified_Prevotellaceae. Mogibacterium* and *Denitratisoma* have been identified as potential causative agents of gingival disease (Liu et al., 2023; Wu et al., 2023). *Mogibacterium* is also associated with intermaxillary gap infections (Sun et al., 2022). *Denitratisoma* is involved in denitrification and are used in sewage treatment (Wang et al., 2023), but their role in the gut requires further investigation. *Catenisphaera* and *Syntrophococcus* are typically associated with the synthesis of short-chain fatty acids and gut protection (Horvath et al., 2020; Yang et al., 2022). However, they were enriched in the D-galactose group. It is hypothesized that this may be due to a specific competitive advantage in this animal model or the disruption of intestinal homeostasis under certain conditions. *Alloprevotella, Colidextribacter, Lachnospira*, and *Lachnospiraceae_ND3007_group* are associated with anti-inflammation and lipid metabolism (Leibovitzh et al., 2022; Xie et al., 2022; Hou et al., 2023). They are involved in combating oxidative stress and are often considered probiotic. *Oscillospira* is seen as a possible next-generation probiotic, a sign of intestinal health, and a producer of short-chain fatty acids (Yang et al., 2021). The *unclassified Erysipelotrichaceae* family is involved in lipid metabolism and promotes piglet weight gain (Tang et al., 2024). The role of *Prevotella*, *Ruminococcus,* and *Schwartzia*is debated and may be related to different strain. The *Eubacterium xylanophilum* group is typically regarded as a harmful bacterium and has been linked to metabolic diseases and colon cancer (Li et al., 2020; Li H. et al., 2022). The divergent microorganisms upregulated by PQ or D-galactose treatment were strongly correlated with inflammation and oxidative stress, according to subsequent correlational analyses. Conversely, negative correlations were observed for these downregulated microbes. These findings imply that, in weaned pigs under oxidative stress, alterations in the microbe composition can be connected to antioxidant decompensation and inflammatory responses.

Despite the extensive use of the acute and chronic oxidative stress piglet model, there are still some limitations to be considered. The construction of an acute oxidative stress model necessitates the use of paraquat, which is banned in numerous countries and difficult to obtain. Consequently, paraquat administration inevitably causes lung damage and renders the model distinct from the actual pathological state. Furthermore, the construction of a chronic oxidative stress model is more resource-intensive in terms of personnel, material resources, and time. Finally, the intestinal molecular mechanisms involved in acute and chronic oxidative stress modeling remain incompletely understood.

In summary, the two types of oxidative stress can systematically damage the growth performance, antioxidant capacity, inflammation response of weaned piglets, while D-galactose as chronic stress can cause severe redox dysregulation, paraquat as acute stress can easily lead to inflammation and liver damage. Furthermore, chronic or acute oxidative stress can disrupt the dynamic balance of intestinal flora, reducing their diversity and composition. However, the structure of microbes in acute and chronic oxidative stress models differs, indicating that the pathological states or molecular pathways involved in these two models may vary, so further studies are needed to understand the mechanism by gut-liver axis and gut microbiota. These results provide valuable insights and direction for future work exploring the relationship oxidative stress and their associated pathologies.

## Data availability statement

The datasets presented in this study can be found in the Genome Sequence Archive (GSA), https://bigd.big.ac.cn/gsa/browse/CRA015743, under accession numbers CRA015743 and CRA015743.

## Ethics statement

The animal study was approved by Animal Welfare Committee of the Institute of Subtropical Agriculture, Chinese Academy of Sciences. The study was conducted in accordance with the local legislation and institutional requirements.

## Author contributions

HZ: Data curation, Formal analysis, Investigation, Methodology, Visualization, Writing – original draft, Writing – review & editing. XuaX: Formal analysis, Investigation, Methodology, Visualization, Writing – original draft, Data curation. CW: Investigation, Methodology, Writing – original draft. TL: Conceptualization, Project administration, Resources, Supervision, Validation, Writing – review & editing. XupX: Project administration, Resources, Supervision, Validation, Writing – review & editing. LH: Conceptualization, Formal analysis, Funding acquisition, Project administration, Resources, Supervision, Validation, Visualization, Writing – review & editing, Data curation.

## Footnotes

^1^http://www.biocloud.net/

^2^http://bigd.big.ac.cn/gsa

## Glossary

ROS: Reactive oxygen species
BW: Body weight
ADFI: Average daily feed intake
ADG: Average daily gain
FCR: Feed conversion ratio
DR: Diarrhea rate
TP: Total protein
IgG: Immunoglobulin G
IgM: Immunoglobulin M
ALT: Alanine aminotransferase
AST: Aspartate aminotransferase
ALP: Alkaline phosphatase
LDH: Lactate dehydrogenase
MDA: Malondialdehyde
SOD: Superoxide dismutase
T-AOC: Total antioxidant capacity
CAT: Catalase
GSH: Glutathione
GSH-Px: Glutathione peroxidase
GSSG: Oxidized glutathione
IgA: Immunoglobulin A
L-1β: Interleukin 1-β
IL-10: Interleukin-10
IL-12: Interleukin-12
TNF-α: Tumor necrosis factor-α
IFN-γ: Interferon-γ
iFABP: Intestinal fatty acid binding protein
DAO: Diamine oxidase
ATP: Adenosine triphosphate
V/C: Villus height to crypt depth ratio
CAR: Constitutive androstane receptor
OTUs: Operational taxonomic units
GCL: Glutamate cysteine ligase
CCRP: Cytoplasmic CAR retention protein
PP2A: Protein phosphatase 2A
